# Geriatric assessment for older patients with breast cancer: A single-institution study

**DOI:** 10.3389/fonc.2023.1031682

**Published:** 2023-02-23

**Authors:** Yan Lin, Ying Xu, Changjun Wang, Yu Song, Yali Xu, Xiaohui Zhang, Xin Huang, Qiang Sun

**Affiliations:** Department of Breast Disease, Peking Union Medical College Hospital, Peking Union Medical College, Beijing, China

**Keywords:** breast cancer, older patients, geriatric analysis, survival rate, survival model

## Abstract

**Introduction:**

Although geriatric assessment (GA) has been used for a long time in the field of geriatrics and internal medicine, there are few studies on its application in the field of breast surgery. Therefore, the utility of specific GA domains for the assessment of older patients with breast cancer remains unclear. The aim of the present study was to evaluate the association between specific GA domains and the survival rate of older patients with breast cancer.

**Methods:**

We used the database of Peking Union Medical College Hospital to identify older patients who were newly diagnosed with breast cancer between 2012 and 2018 and retrospectively analysed the data of 541 patients aged ≥65 years. Patients with metastatic cancer and those with missing vital status data were excluded. The primary outcomes were overall survival (OS) and breast cancer-specific survival. The GA domains used in this study included functional status, comorbidities, and psychological state. Multivariate regression analysis was used to estimate hazard ratios for these three domains.

**Results:**

After a median follow-up of 72 months, we observed a significant relationship between functional impairment and mortality (adjusted HR: 3.06, 95% confidence interval [CI]: 1.83-5.10, P<0.001). Similarly, patients with severe comorbidities (adjusted HR: 2.35; 95% CI: 1.16-4.75, P=0.017) and an impaired psychological state (adjusted HR: 2.82, 95% CI: 1.45-5.50, P=0.002) showed worse OS rates. Accordingly, addition of the three GA domains to the basic model, which included age, tumour stage, lymph node stage, and intrinsic molecular subtype as baseline variables, yielded higher C‐statistics for mortality analysis (from 0.713 to 0.740).

**Conclusion:**

To our knowledge, this is the first study to include specific GA domains in a prognostic model for older patients with breast cancer in China. Three domains, namely functional status, comorbidities, and psychological state, should be considered for survival analyses in this particular population. The full model including these three GA domains may be more accurate in predicting the survival of older patients with breast cancer.

## 1 Introduction

The number of older patients with cancer has steadily increased over the past10 years, with the progress of social aging (1). In addition, the prevalence of breast cancer in older adults (≥65 years) has been increasing of late (2). Cancer characteristics and treatment responsiveness tend to differ between older and younger patients with breast cancer (3). Therefore, the management of breast cancer in older patients is challenging because the disease is highly heterogeneous, and there is insufficient evidence specific to older adults. This can complicate the clinical decision-making process. The use of chronological age alone to determine treatment strategies increases the risk of overtreatment or undertreatment in older patients (4). Decision-making should involve use of the geriatric assessment (GA) and also consider patient preferences (5). Therefore, it is important to identify age‐related prognostic factors, such as comorbidities and functional status, that can support survival predictions and treatment decisions.

GA is believed to facilitate better estimations of life expectancy and assist treatment decisions in the field of geriatric oncology. GA typically comprises several domains, including physical performance, fall risk, functional status, multiple comorbidities, polypharmacy status, depressive symptoms, cognition, psychosocial distress, nutritional status, and socioeconomic support (6). The European Society of Breast Cancer Specialists (EUSOMA) and the International Society of Geriatric Oncology (SIOG) recommend that GA should be used for the management of all older patients with breast cancer (7).

There are several tools for GA, with each comprising different domains (6, 8, 9). Several GA domains have been reported as independent predictors of mortality in patients with cancer (10–13); among these, impaired functional status, comorbidities, and psychological distress are consistently identified as risk factors for mortality. Functional status measures in geriatric oncology commonly involve evaluations of activities of daily living (ADL) and instrumental ADL (IADL); the former encompasses basic self-care skills for independent home‐based living and is more representative and illustrative (14). Comorbidities refer to one or more disorders in addition to the specific cancer. These conditions become increasingly prevalent with advancing age and are associated with poorer outcomes in older patients with cancer (15). Emerging evidence also suggests that psychological distress is strongly correlated with cancer mortality in older patients (16). However, the mechanisms underlying psychological challenge-mediated tumour immune evasion have not been systematically explored.

Although there is increasing information on the impact of various GA domains on overall survival (OS) in patients with cancer, there is little relevant research involving older patients with breast cancer. Large-scale studies are warranted for more accurate identification of crucial GA domains that would assist survival predictions and treatment choices for this patient population. Accordingly, the aim of this retrospective study was to evaluate the associations of three GA domains, namely functional status, comorbidities, and psychological state, with survival in older patients with breast cancer.

## 2 Materials and methods

### 2.1 Study design and patient population

The Peking Union Medical College Hospital (PUMCH) database has been collecting the data of patients with breast cancer who have undergone treatment at PUMCH since 1975. These data includes patient age at cancer diagnosis, sex, vital status, surgical methods, tumour histology, treatment regimens, concomitant diseases, details regarding recurrence or metastasis (localised, regional, and distant), and survival information. For the present study, any hard copies of patient records were electronically filed by scanning. The necessary data were extracted and input into a new database to enable analysis of the associations between clinical information and mortality.

Eventually, data for 541 patients aged ≥65 years were collected. All patients underwent surgery and other treatments from 2012 to 2018 and underwent clinical follow-up at PUMCH. Written informed consent to undergo the procedure and follow-up assessments was obtained from all patients. The study was approved by the Institutional Review Board of Peking Union Medical College Hospital (ZS-2682) and performed in accordance with the principles of the 1964 Declaration of Helsinki and its later amendments or comparable ethical standards. Because the study was retrospective in nature and all patient data were anonymised, the need for informed consent for publication of this report was waived.

We first identified patients aged ≥65 years (n = 622) who were diagnosed with breast cancer between 1 January 2012 and December 2018. We used a cut-off age of 65 years because it is frequently designated as the age for GA implementation in geriatric oncology studies. At the same time, clinical trials related to breast cancer have often selected 65 years as the cut-off age to distinguish elderly patients. Patients with incomplete basic information (n = 38), missing vital status details (n = 42), or simultaneous metastatic cancer (n = 11) were excluded. All patients underwent monitoring to verify their vital status using the PUMCH follow-up system until December 2021.

### 2.2 Assessment of GA domains

Three specific GA domains, namely functional status, comorbidities, and psychological state, were used for assessments in this study. The patients admitted to our surgery ward are all ready for breast surgery; in other words, we tend to screen relatively ‘healthy’ older patients in our ward. Therefore, we first have to collect functional status and comorbidity data in the outpatient department and then collect psychological data; these data are supplied after admission of the patient. Data collection in the outpatient department, rather than administration of the entire GA questionnaire, facilitates a convenient and effective method for input.

Functional status was assessed using the Barthel Index score, which includes 10 items to measure ADL performance (17). These items include continence and independence in bathing, feeding, dressing, using the bathroom, getting up, and moving around the house. A total score ranging from 0 to 100 is calculated for each patient, with higher scores indicating higher levels of independence. We recorded the functional status as abnormal (0‐60) or normal (61‐100) based on the information provided by the patients. If the patient could not provide any detail precisely, we marked the status as unknown.

Comorbidities were assessed using the Charlson Comorbidity Index (CCI) score, which includes 17 comorbid conditions (myocardial infarction, congestive heart failure, peripheral vascular disease, cerebrovascular disease, dementia, chronic pulmonary disease, rheumatologic disease, peptic ulcer disease, liver disease, diabetes, hemiplegia or paraplegia, renal disease, any malignancy, and human immunodeficiency virus [HIV] infection) (18). From these, HIV infection was excluded because affected patients require treatment at designated hospitals. Furthermore, metastatic cancer was an exclusion criterion in the present study, whereas patients with paraplegia generally opt for drugs rather than surgery. Therefore, only 14 comorbid conditions were considered in the present study. There are four kinds of weights for each comorbid condition (1, 2, 3, and 6) based on the mortality risk associated with that condition. The following three categories were used for comorbidities in the present study: none (CCI score: 0–1), mild-to-moderate (CCI score: 2–3), and severe (CCI score: ≥4).

Psychological state was assessed using the updated Geriatric Depression Scale (GDS-15), which includes 15 items and is used to diagnose and evaluate depression in elderly individuals (19, 20). Psychological state was categorised as abnormal or depressive state (GDS ≥ 8), normal or non-depressive state (GDS < 8), and unknown.

### 2.3 Outcome variables

The primary outcomes were OS and breast cancer-specific survival (BCSS), estimated from the time of GA. We obtained detailed mortality data from our study database and retrieved the causes of death for all patients who died during the study period. Mortality was classified as BCSM and all-cause mortality.

### 2.4 Statistical analyses

Categorical variables are expressed as number and percentage. Pearson’s chi-square test was used to compare the results between the different groups. Continuous variables are presented as median with interquartile range.

A Cox proportional hazards model was constructed to estimate the independent effects of the three GA domains on the survival rate during a 6-year follow-up period, after adjustment for age, tumour stage, lymph node (LN) stage, and intrinsic molecular subtype. The primary outcome was OS time, which was defined as the time from the date of cancer diagnosis to the date of death from any cause or the date on which the patient was last known to be alive. The estimated effects of the three GA domains on OS were calculated as hazard ratios (HRs) and 95% confidence intervals (CIs). Adjusted survival curves stratified by the categories of the three GA domains were also generated. To examine the effects of the three GA domains on OS and BCSS, we constructed a Cox proportional hazards model after adjusting for age, tumour stage, LN stage, and intrinsic molecular subtype.

To assess the incremental prognostic value of each GA domain, we estimated Harrell’s concordance statistic (C‐statistic) for different models for OS and BCSS. The C‐statistic is equivalent to the area under the receiver operating characteristic curve, with a value of 0.5 indicating random predictions and a value of 1.0 indicating perfect discrimination between survivors and non-survivors. The first model was a ‘basic’ model that controlled for age, tumour stage, LN stage, and intrinsic molecular subtype. The GA domains were then individually added to the basic model. The final model was a ‘full’ model that included all three GA domains in addition to the covariates in the basic model.

All analyses were conducted using R software version 4.1.3. A two-sided P-value of <0.05 was considered statistically significant.

## 3 Results

### 3.1 Patient characteristics

**Figure 1:**
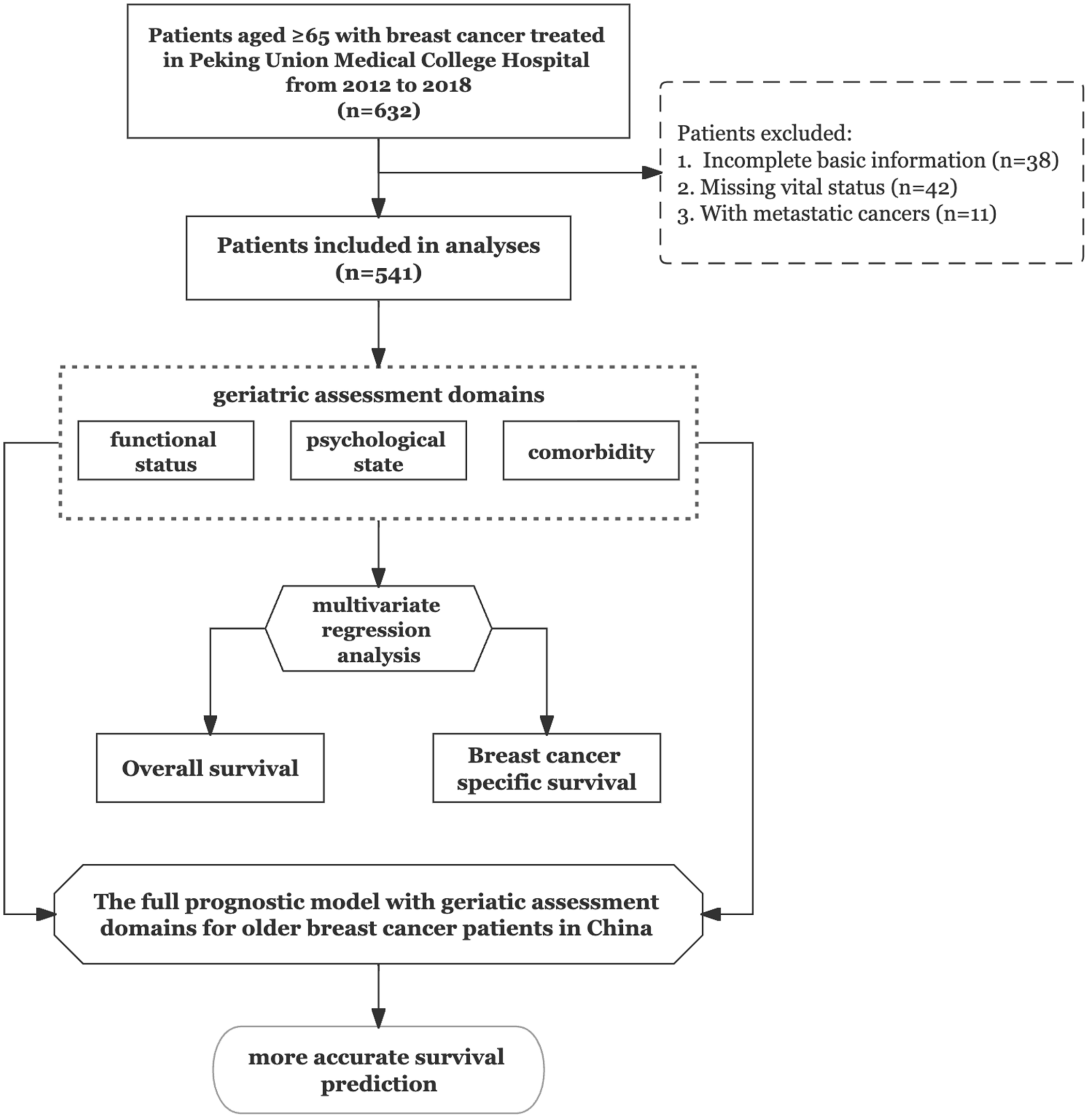
Study flowchart.

The study flowchart is shown in **Figure 1**. We recruited older patients (≥65 years) who underwent breast surgery at our cancer centre. From the 632 patients considered eligible, we identified 541 patients as study subjects. Patient characteristics are listed in **Table 1**. Of the 541 patients, 169 (31%) were aged ≥75 years at the time of cancer diagnosis. The most common cancer subtype was luminal B, and the most common tumour stage was stage II. During the 6‐year follow‐up period, the all-cause mortality rates for the <75-year and ≥75-year groups were 11.3% and 24.9%, respectively, with a significant difference between groups. There was no significant difference in the BCSM rate between the two groups. We also observed significant difference in functional status between the two groups, with no significant differences in the distributions of intrinsic molecular subtype, tumour stage, LN stage, comorbidities, and psychological state.

**Table 1:** Patient and tumour characteristics in groups stratified by age.

	≤75y (%)	>75y (%)	*P* value
**Total number of patients**	372 (69.0)	169 (31.0)	
**All-cause mortality**	42 (11.3)	42 (24.9)	**<0.001**
**Breast cancer specific mortality**	28 (7.5)	13 (7.7)	0.852
**Intrinsic Molecular Subtype**			0.731
** Luminal A**	82 (22.0)	33 (19.5)	
** Luminal B**	155 (41.7)	76 (45.0)	
** HER-2 enriched**	23 (6.2)	8 (4.7)	
** Basal-like**	46 (12.4)	18 (10.7)	
** Unknown**	20 (5.4)	12 (7.1)	
**Tumor Stage**			0.719
** 0**	46 (12.4)	22 (13.0)	
** I**	185 (49.7)	89 (52.7)	
** II**	130 (34.9)	51 (30.2)	
** III**	8 (2.2)	5 (3.0)	
** Unknown**	3 (0.8)	2 (1.2)	
**LN Stage**			0.094
** 0**	95 (25.5)	41 (24.3)	
** I**	36 (9.7)	10 (5.9)	
** II**	13 (3.5)	12 (7.1)	
** III**	25 (7.8)	7 (21.9)	
** unknown**	203 (54.6)	99 (58.6)	
**Functional Status (ADL)**			**<0.001**
** Normal**	338 (90.9)	125 (74.0)	
** Abnormal**	30 (8.1)	34 (20.1)	
** Unknown**	4 (1.0)	10 (5.9)	
**Comorbidities(CCI score)**			0.992
** None**	71 (19.1)	32 (18.9)	
** Mild-to-moderate**	207 (55.6)	95 (56.2)	
** Severe**	94 (25.3)	42 (24.9)	
**Psychological state**			0.466
** Normal**	352 (94.6)	156 (92.3)	
** Abnormal**	17 (4.6)	12 (7.1)	
** Unknown**	3 (0.8)	1 (0.6)	

ADL, activities of daily living; LN, lymph node; HER2, human epidermal growth factor receptor 2; CCI, Charlson Comorbidity Index. The bold P values listed were recognized as statistically significant.

### 3.2 Survival data for the GA domains

Multivariate Cox proportional hazards models were used to assess all-cause mortality and BCSM according to the three GA domains. HRs were calculated using Cox proportional hazards models adjusted for age, tumour stage, LN stage, and intrinsic molecular subtype. **Table 2** presents the results.

**Table 2:** Adjusted HRs for all-cause mortality and BCSM based on geriatric assessment domains.

	All-cause mortality			BCSM		
	number of events (%)	Adjusted HR (95%CI)	*P*	number of events (%)	Adjusted HR (95%CI)	*P*
Functional Status						
** Normal**	56 (12.1)	1	–	31 (6.7)	1	–
** Abnormal**	25 (39.1)	3.06 (1.83-5.10)	**<0.001**	8 (12.5)	2.22 (0.98-4.98)	0.054
** Unknown**	3 (21.4)	0.92 (0.27-3.18)	0.901	2 (14.3)	3.26 (0.67-15.9)	0.144
Comorbidities						
** None**	13 (12.6)	1	–	8 (7.8)	1	–
** Mild-to-moderate**	43 (14.2)	1.59 (0.84-3.04)	0.156	23 (7.6)	1.64(0.69-3.89)	0.260
** Severe**	28 (20.6)	2.35 (1.16-4.75)	**0.017**	10 (7.4)	1.94 (0.71-5.27)	0.196
Psychological state						
** Normal**	72 (85.7)	1	–	38 (7.4)	1	–
** Abnormal**	11 (13.1)	2.82 (1.45-5.50)	**0.002**	3 (10.3)	1.56 (0.45-5.37)	0.480
** Unknown**	1 (1.2)	1.71 (0.23-13.02)	0.603	0 (0.0)	–	–

HR, hazards ratio; CI, confidence interval; BCSM, breast cancer-specific mortality. The bold P values listed were recognized as statistically significant.

In the all‐cause mortality analysis, we observed a significant relationship between ADL impairment and mortality. Breast cancer patients with abnormal ADL had a significantly higher risk of all-cause mortality (adjusted HR: 3.06, 95% CI: 1.83-5.10, P<0.001) than did functionally independent patients. There was no significant difference in BCSM between patients with ADL impairment and those without ADL impairment (adjusted HR: 2.22, 95% CI: 0.98-4.98, P=0.054).

Meanwhile, the models for comorbidities and psychological state yielded similar results for geriatric impairment and mortality. Patients with severe comorbidities had a significantly higher hazard of all-cause mortality (adjusted HR: 2.35, 95% CI: 1.16-4.75, P=0.017) than did those without comorbidities. With regard to BCSM, there was no significant difference between patients with severe comorbidities and those with no comorbidity after adjustment (adjusted HR: 1.94, 95% CI:0.71-5.27, P=0.196). This was also observed in the analysis of psychological state. Relative to normal patients, patients with psychological abnormalities had an adjusted HR of 2.82 (95% CI: 1.45-5.50, P=0.002) for all-cause mortality. For BCSM, there was no significant difference between normal patients and patients with psychological abnormalities (adjusted HR: 1.56, 95% CI: 0.45-5.37, P=0.480).

**Figure 2:**
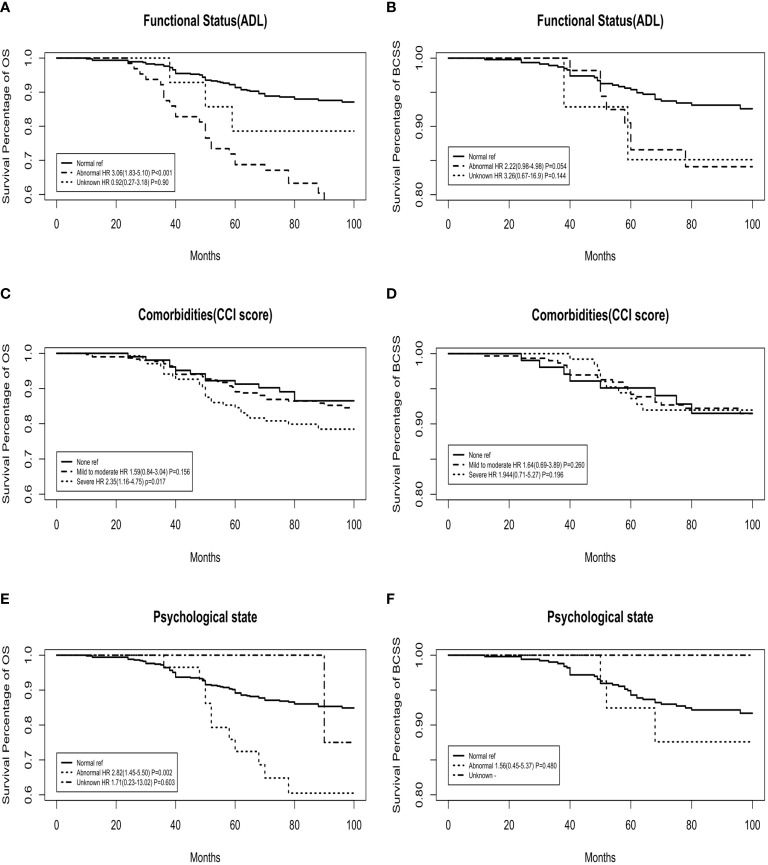
Adjusted overall survival (OS) and breast cancer-specific survival (BCSS) curves for functional status, comorbidities, and psychological state domains of the geriatric assessment. **(A, C, E)** OS curves; **(B, D, F)** BCSS curves.

**Figure 2** shows the OS and BCSS curves for each GA domain after adjusting for age, tumour stage, LN stage, and intrinsic molecular subtype. OS was significantly higher for patients with an inferior geriatric status. In BCSS analysis, there was no significant relationship with the GA domains.

The OS rate was significantly lower in the abnormal function group than in the normal function group (adjusted HR: 3.06, 95% CI: 1.83‐5.10, P<0.001), while the BCSS analysis showed no significant interaction between these two groups. The comorbidity model yielded results that were similar to those of the functional model. The OS rate for patients with none‐to‐moderate comorbidities was higher than that for patients with severe comorbidities (for severe comorbidities, adjusted HR: 2.35, 95% CI: 1.16‐4.75, P=0.017). Similarly, there was a significant difference in HR according to the psychological state. All-cause mortality was significantly higher in patients with an abnormal psychological state (adjusted HR: 2.82, 95% CI: 1.45‐5.50, P=0.002). However, BCSS analysis showed no significant interaction between these two groups (adjusted HR: 1.56, 95% CI: 0.45‐5.37, P=0.480).

### 3.3 Survival data for tumour domains

The results of mortality analyses for patients stratified by tumour stage, LN stage, and intrinsic molecular subtype are shown in **Table 3**.

**Table 3:** Adjusted HRs for all-cause mortality and BCSM according to tumour stage, LN stage, and subtype.

	All-cause mortality			Breast cancer specific mortality		
	Mortality (%)	Adjusted HR (95% CI)	*P*	Mortality (%)	Adjusted HR (95% CI)	*P*
Tumor Stage						
** 0**	6(7.1)	1		1(2.4)	1	–
** I**	40(47.6)	1.96(0.81-4.72)	0.133	15(36.6)	4.10(0.54-31.18)	0.172
** II**	33(39.3)	2.34(0.96-5.70)	0.062	22(53.7)	9.12(1.22-67.99)	**0.031**
** III**	4(4.8)	6.36(1.74-23.25)	**0.005**	2(4.9)	14.87(1.32-166.85)	**0.029**
** Unknown**	1(1.2)	3.45(0.41-29.30)	0.257	1(2.4)	15.7(0.97-255.95)	0.053
LN Stage						
** 0**	19(22.6)	1		9(22.0)	1	
** I**	8(9.5)	1.20(0.52-2.75)	0.667	7(17.1)	2.25(0.83-6.08)	0.110
** II**	3(3.6)	0.82(0.24-2.78)	0.751	0(0.0)	–	–
** III**	12(14.3)	2.98(1.43-6.19)	**0.004**	12(29.3)	6.61(2.74-15.92)	**<0.001**
** Unknown**	42(50.0)	0.86(0.49-1.49)	0.585	13(31.7)	0.58(0.24-1.38)	0.216
Subtype						
** Luminal A**	10(11.9)	1		4(9.8)	1	
** Luminal B**	33(39.3)	1.20(0.43-3.37)	0.725	15(36.6)	2.80(0.31-25.24)	0.359
** HER-2 enriched**	7(8.3)	1.82(0.75-4.41)	0.183	6(14.6)	4.95(0.65-37.61)	0.122
** Basal-like**	18(21.4)	3.57(1.17-10.90)	**0.026**	12(29.3)	18.04(2.13-152.94)	**0.008**
** Unknown**	10(11.9)	4.66(1.81-12.00)	**0.001**	3(7.3)	18.71(2.39-146.35)	**0.005**

HR, hazards ratio; CI, confidence interval; BCSM, breast cancer-specific mortality; LN, lymph node; HER2, human epidermal growth factor receptor 2. The bold P values listed were recognized as statistically significant.

After adjustment for ADL, CCI, and the psychological state, stratifications of these three tumour domains markedly affected the results for both all-cause mortality and BCSM.

Specifically, compared with other tumour stages, stage III was significantly associated with higher all-cause mortality (adjusted HR: 6.36, 95% CI: 1.74‐23.25) and BCSM (adjusted HR: 1.51, 95% CI: 1.34‐1.71). Likewise, patients with stage 3 LNs showed a higher risk of all-cause mortality (adjusted HR: 2.98, 95% CI: 1.43‐6.19) and BCSM (adjusted HR: 6.61, 95% CI: 2.74‐15.92). With regard to the intrinsic molecular subtype, the basal-like subtype was significantly associated with higher all-cause mortality (adjusted HR: 3.57, 95% CI: 1.17‐10.90) and BCSM (adjusted HR: 18.04, 95% CI: 2.13‐152.94). However, the unknown group was associated with lower mortality.

### 3.4 Estimates for the prognostic model

The incremental prognostic value of the three GA domains in our statistical models was analysed by comparing the C-statistics of the different models for all-cause mortality and BCSM (**Table 4**). The basic model included the baseline variables of age at diagnosis, tumour stage, LN stage, and intrinsic molecular subtype. The full model included functional status, comorbidities, and psychological status in addition to the baseline variables.

**Table 4:** Comparison of model performance for all-cause mortality and BCSM.

	Harrell’s concordance statistic	
	All-cause mortality	BCSM
Basic model	0.713(se=0.003)	0.775(se=0.039)
Basic model+ADL	0.731(se=0.003)	0.782(se=0.039)
Basic model+CCI	0.722(se=0.003)	0.776(se=0.039)
Basic model+Psychological status	0.728(se=0.030)	0.779(se=0.038)
Basic model+ADL+CCI	0.732(se=0.031)	0.781(se=0.039)
Basic model+ADL+ Psychological status	0.740(se=0.030)	0.783(se=0.039)
Basic model+CCI+ Psychological status	0.732(se=0.030)	0.781(se=0.038)
Full model	0.740(se=0.030)	0.783(se=0.039)

SE, standard error; BCSM, breast cancer-specific mortality; ADL, activities of daily living; CCI, Charlson Comorbidity Index.

Compared with the basic model, models that added any of the three GA domains showed higher C‐statistics for all-cause mortality and BCSM. Among the three GA domains, functional status facilitated the largest increase in model performance. The full model with all three GA domains yielded the highest C‐statistics for both all-cause mortality and BCSM.

## 4 Discussion

For older patients with breast cancer, oncologists have to determine the eligibility of patients to receive treatment according to the general guidelines; furthermore, they must identify patients who require surveillance because of poor tolerance. Previous studies have demonstrated that age alone cannot determine treatment strategies for older patients with cancer (21), and the implementation of comprehensive GA involving numerous domains has been recommended by EUSOMA and SIOG (7). However, comprehensive GA is time-consuming, and not all domains have been validated in terms of breast cancer treatment (22). To our knowledge, this is the first study in China to focus on the prognostic value of functional status, comorbidities, and psychological state for survival analyses of older patients with breast cancer. Our findings support the utility of these GA domains for achieving improved prognostic accuracy and making informed treatment decisions for older patients with breast cancer.

In the present study, functional status, comorbidities, and psychological state were independently associated with the OS rate after adjustment for clinical variables commonly used by oncologists in the risk assessment of BCSM (age, tumour stage, LN stage, and intrinsic molecular subtype).

The Eastern Cooperative Oncology Group Performance Status (ECOG PS) is the most widely used functional assessment tool in oncology. However, several studies have demonstrated that ECOG PS may underestimate the degree of functional impairment, particularly in older patients with cancer (23). Therefore, ADL evaluation tools such as the Barthel Index are recommended as a better alternative by EUSOMA and SIOG (24). In the present study, we found that ADL impairment at the time of cancer diagnosis was associated with poorer OS; this was consistent with the findings of previous clinical trials (22, 25). According to our findings, there was no significant association between the ADL score and BCSS. In addition, we determined an association between ADL impairment and poorer OS, which can be explained by the increased risk of mortality associated with their general health status, lower treatment feasibility, and increased treatment-related adverse reactions.

In addition, the prognostic impact of comorbidities was observed in this study. While the presence of comorbidities was associated with a poorer OS rate, it was not associated with the BCSS rate, probably because of the increased risk of mortality from concurrent diseases, which can be considered to have a direct effect on OS. The coexistence of breast cancer with another disease affects the treatment of both conditions; this is also an important reason for the lower OS rate (26). Our findings support the belief that comorbidities are an essential component of GA, as observed in other relevant studies (27–29).

Psychological state is another important factor in terms of the prognosis and treatment decision for breast cancer, particularly that in elderly patients (30–32). Our finding of a significant association between an impaired psychological state at cancer diagnosis and poorer OS is concordant with the findings in previous studies (31–33). As observed with the other two GA domains, this association was observed only with OS, not BCSS. A possible explanation for this finding is that the psychological state affects overall immune function in an individual; therefore, it is significantly related to OS, which is associated with all concurrent diseases (34, 35).

In addition to focusing on the above GA domains, the present study also demonstrated the prognostic impact of tumour-related domains in older patients with breast cancer. Several clinical studies have confirmed that tumour-related factors such as tumour stage, LN stage, and intrinsic molecular subtype are significant independent factors for the prognosis of elderly patients with breast cancer (24, 36, 37). The present study yielded consistent results for both OS and BCSS.

Furthermore, we evaluated the prognostic value of a novel competing risk approach including the three GA domain and tumour-related variables and showed the incremental prognostic value of functional status, comorbidities, and psychological status by adding them to a basic statistical model including age, tumour stage, LN stage, and intrinsic molecular subtype as baseline variables. The full model with all three GA domains significantly increased the prediction ability of the basic statistical model; this indicates that comprehensive consideration of tumour factors and GA domains is valuable for predicting the prognosis and deciding the treatment of elderly patients with breast cancer.

### 4.1 Limitations

This study has several limitations. First, we extracted clinical information from a data source at a single cancer centre, which was unable to provide extensive and universal clinical information. Therefore, our study population may not be representative of the general population. Second, the psychological state at cancer diagnosis may be an inaccurate indicator because it is a subjective domain, particularly for elderly patients with cancer. However, this is a common problem in clinical psychological evaluation. Third, because of the lack of previous research on the utility of GA for elderly patients with breast cancer, we selected the three GA domains based on data for other cancer types. Accordingly, selection bias could not be avoided because we were unable to examine other GA domains. Fourth, we simplified the grades for ADL and CCI to adapt to the clinical application of surgery; thus, the indicators may not be sufficiently precise. However, implementation of the original form of GA would be too cumbersome and not conducive to clinical implementation. Future studies are warranted for the development of an algorithm with improved accuracy for convenient clinical applications. Fifth, the present study only focused on the relationship between GA domains and prognosis, and the applicability and effectiveness of GA domains in terms of treatment options remain unclear and will be the scope of our future research.

## 5 Conclusion

In the present study, we validated the association between three specific GA domains and OS in older patients with breast cancer and found that addition of functional status, comorbidities, and psychological state to a basic model including tumour-relevant variables was valuable and useful for comprehensive assessments to predict long-term survival in older patients with breast cancer. Although further studies are needed to verify the contribution of these GA domains to the treatment decision-making process, this predictive model should be considered when discussing the risks and benefits of clinical intervention for older patients with breast cancer.

## Data availability statement

The raw data supporting the conclusions of this article will be made available by the authors, without undue reservation.

## Ethics statement

The studies involving human participants were reviewed and approved by the Institutional Review Board of the Peking Union Medical College Hospital. The patients/participants provided their written informed consent to participate in this study.

## Author contributions

YL and YX: conceptualisation, methodology, formal analysis, writing, editing, and review. YS: conceptualisation, methodology, investigation, data curation, and writing. YLX: methodology, investigation, and resources. CJW: investigation, resources, and editing. XZ: methodology and review. XH: methodology and review. QS: conceptualisation, methodology, review, project administration, and funding acquisition. All authors contributed to the article and approved the submitted version.
